# Reversible Bilateral Lower Extremity Weakness Secondary to Severe Hypomagnesemia Following Acute Diarrheal Illness

**DOI:** 10.7759/cureus.81077

**Published:** 2025-03-24

**Authors:** Marcos Molina, Raydi Mejia Landron, Fouad Kaddour-Hocine, Neguemadjii Ngardig Ngaba, Rosanna Pineda, Misbahuddin Khaja

**Affiliations:** 1 Internal Medicine, BronxCare Health System, New York City, USA

**Keywords:** diarrheal illness, electrolyte disturbance, hypomagnesemia, lower extremity weakness, neuromuscular manifestations

## Abstract

Hypomagnesemia is a common electrolyte abnormality that can cause a wide range of neuromuscular symptoms, including muscle weakness, tremor, and tetany. However, it is a rare cause of isolated lower extremity weakness. We present a case of a 71-year-old female with multiple comorbidities who developed severe bilateral lower extremity weakness and upper extremity tremor in the setting of profound hypomagnesemia (serum magnesium level of 1.0 mg/dL) after an acute diarrheal illness. The patient's weakness and tremor rapidly improved with intravenous magnesium supplementation. This case highlights the importance of considering hypomagnesemia in the differential diagnosis of acute bilateral lower extremity weakness and the potential for rapid reversal with magnesium repletion.

## Introduction

Lower extremity weakness has broad differential causes, including neurological, musculoskeletal, infectious, inflammatory, rheumatologic, genetic, drug-induced, electrolyte imbalances, and metabolic etiologies [1]. Electrolyte abnormalities, particularly hypokalemia and hypophosphatemia, are well-recognized causes of generalized weakness [2]. Hypomagnesemia, a common electrolyte imbalance affecting 7-11% of hospitalized patients, can trigger various neuromuscular symptoms, including tremor, tetany, seizures, and cardiac arrhythmias. These manifestations primarily result from secondary hypocalcemia that develops when serum magnesium levels fall below 1.2 mg/dL (1.6-2.6 mg/dL) [3-5]. However, isolated lower extremity weakness as the primary presenting symptom of hypomagnesemia is uncommon. We report a case of severe hypomagnesemia causing acute bilateral lower extremity weakness and tremor following an episode of acute diarrhea.

## Case presentation

A 71-year-old woman with a history of coronary artery disease status post-coronary artery bypass grafting, hypertension, diabetes mellitus, atrial fibrillation on anticoagulation, hypothyroidism, gastro-esophageal reflux disease on daily proton pump inhibitor and peripheral neuropathy presented with progressive bilateral lower extremity weakness, upper extremity tremor, and non-exertional chest pain. Three days prior to presentation, she had developed severe watery diarrhea lasting approximately 10 hours and had decreased appetite. After the resolution of her diarrhea, she noticed generalized weakness, greater in the lower extremities proximally, to the point that her knees would buckle when trying to walk as well as tremors in her upper extremities.

On examination, vital signs were stable. Cardiopulmonary exam was unremarkable. She was alert and oriented to person, place, and time; however, she needed encouragement to maintain the flow of conversation. Neurological exam was notable for bilateral upper extremity postural and action tremor despite normal strength (5/5). Lower extremities revealed symmetric proximal muscle weakness with 3/5 strength in hip flexion and knee extension bilaterally. She was unable to lift her lower extremities off the bed against gravity for more than 5 seconds. Distal lower extremity strength was 4/5 bilaterally. Sensation, including proprioception, pain, temperature, pressure, and vibration, was intact. Deep tendon reflexes were absent in the lower extremities and 1+ in the upper extremities. Plantar responses were flexor. She had no muscle atrophy, fasciculations, or myoclonus. Her initial laboratory evaluation revealed significant hypomagnesemia (1.0 mg/dL) with normal potassium, phosphorus, and calcium levels (3.8 mmol/L, 3.9 mg/dL, and 8.5 mg/dL, respectively).

**Figure 1:**
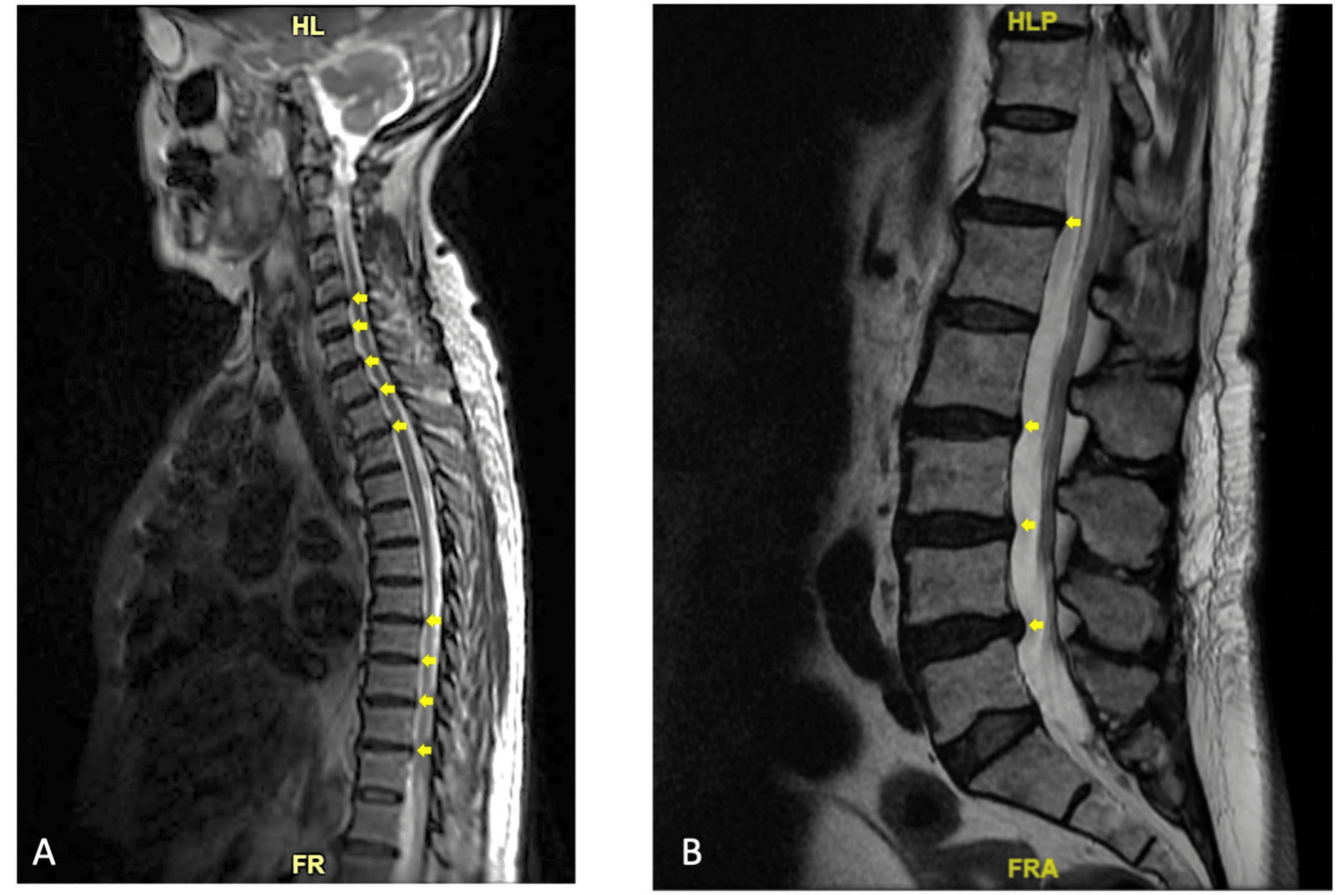
MRI of the thoracic and lumbar spine demonstrated degenerative changes with no evidence of severe spinal canal stenosis. A: MRI thoracic spine; B: MRI lumbar spine; Yellow arrows: small disc bulges.

The patient was admitted for intravenous magnesium sulfate repletion. She received 1 gram of IV magnesium sulfate followed by another 1 gram 1.5 hours later and 2 grams 7 hours later. The following day, her serum magnesium level had increased to 3.1 mg/dL and her tremor and lower extremity weakness were markedly improved. On manual muscle testing, her bilateral lower extremity strength had improved to 5/5 throughout. MRI of the thoracic and lumbar spine showed no acute abnormalities and mild degenerative changes without significant stenosis or cord compression (Figure 1). Lumbar puncture performed on the day of admission was unremarkable and negative for albumino-cytologic dissociation (Table 1 and Table 2). Autoimmune laboratory workup was negative for possible immune-mediated lower extremity weakness such as myositis. Given the patient's symptomatic improvement following magnesium replenishment and the unremarkable initial workup for lower extremity weakness, the neurology service determined that additional diagnostic evaluation was unnecessary at that time and recommended outpatient follow-up monitoring instead. Magnesium was continued at 400 mg PO daily for a total of two days. The etiology of her hypomagnesemia was attributed to gastrointestinal losses related to her acute diarrheal illness, exacerbated by poor oral intake. Upon discharge to a rehabilitation facility two days later, her serum magnesium was stable at 2.6 mg/dL and her lower extremity strength was back to baseline.

**Table 1:** Cerebrospinal fluid (CSF) analysis.

Test	Result	Reference Range
CSF Analysis		
Viral Culture	Negative	Negative
Cytomegalovirus Ab, Quantitative DNA-PCR (CSF)	Not Detected	(log IU/mL)
VDRL, CSF Quantitative	Nonreactive	Nonreactive
Multiple Sclerosis Panel		
Oligoclonal Bands, CSF	Absent	Absent
Synthesis Rate IgG, CSF	-5.3 mg/24h	-9.9-3.3 mg/24 h
IgG Index CSF	0.45	(<70)
Albumin, CSF	21.1 mg/dl	(8.0-42.0)
Immunoglobulin G, CSF	4.2 mg/dl	(0.8-7.7 mg/dL)
Immunoglobin G Level, Serum	1430 mg/dl	(600-1540 mg/dL)
CSF Bacterial Antigen		
Haemophilus influenzae B	Negative	Negative
Streptococcus pneumoniae	Negative	Negative
Group B Streptococcus	Negative	Negative
Neisseria meningitidis C/W135	Negative	Negative
Neisseria meningitidis A/Y	Negative	Negative
Neisseria meningitidis B/E coli K1	Negative	Negative
Cryptococcal Antigen	Not Detected	Not Detected
Mycobacteria Culture w/Fluorochrome, CSF	No acid-fast bacilli seen using the fluorochrome method	-
Miscellaneous Fungal Culture	No fungal growth at 4 Weeks	-
Gram Stain	No organisms seen	-
Aerobic Culture, CSF	No Growth	No Growth
Glucose CSF	100 mg/dl	(40-70 mg/dl)
Protein CSF	37 mg/dl	(1.5-45 mg/dl)
Lactic Acid Dehydrogenase, CSF	23	-
Cell Count + Differential, CSF		
CSF Color	Colorless	Colorless
CSF Appearance	Clear	Clear
WBC Count, CSF	0	-
RBC Count, CSF	4	-
Total Cells CSF	0	-
Tube Num CSF	1	-

**Table 2:** Additional laboratory results on admission day.

Test	Result	Reference Range
Basic Metabolic Panel		
Sodium	133 mmol/L	(135-145 mEq/L)
Potassium	4.6 mmol/L	(3.5-5.0 mEq/L)
Chloride	104 mmol/L	(98-108 mEq/L)
Blood Urea Nitrogen	29 mg/dL	(6.0-20.0 mg/dL)
Creatinine	1.4 mg/dL	(0.5-1.5 mg/dL)
Calcium	8.9 mg/dL	(8.5-10.5 mg/dL)
Glucose	273 mg/dL	(70-120 mg/dL)
Serum Magnesium	1.0 mg/dL	(1.6-2.6 mg/dL)
Complete Blood Count (CBC)		
Hemoglobin	8.6 g/dL	(12.0-16.0 g/dL)
Other CBC values	Unremarkable	-
Creatine Kinase, Serum	53 unit/L	(20-200 unit/L
Respiratory Viral Panel	Negative	Negative
Thyroid Panel		
Free Thyroxine, Serum	1.45 ng/dL	(0.80-2.00 ng/dL)
T3 (triiodothyronine)	81.1 ng/dL	(60.0-181.0ng/dL)
Thyroid Stimulating Hormone, Serum	2.21 mlU/L	(0.40-4.50 mlU/L)
C3 complement, Serum	160.0 mg/dL	(90.0-150.0 mg/dl)
C4 complement, Serum	60.0 mg/dl	(16.0-47.0 mg/dL)
Rheumatoid Factor, Serum	11.1	(<=14.0 IU/mL)
ANA (antinuclear antibody)	Positive	(Negative)
ANA Titer	0.097222	-
ANA Pattern	Cytoplasmic, Fibrillar	-
ANCA (Antineutrophil Cytoplasmic Antibodies) Vasculitides		
Myeloperoxidase	<1.0	(<1.0)
Proteinase-3 Ab	<1.0	(<1.0)
Sjogren's Antibody		
SS-A antibody	<1.0 NEG	(<1.0)
SS-B antibody	<1.0 NEG	(<1.0)
Anti-DNA Antibody	<1.0 NEG	(<1 IU/mL)
Cardiolipin Antibody Screen		
Cardiolipin Antibody IgA	<2.0 (APL-U/mL)	<20.0 (APL-U/mL)
Cardiolipin Antibody IgM	<2.0 (MPL-U/mL)	<20.0 (MPL-U/mL)
Cardiolipin Antibody IgG	<2.0 (GPL-U/mL)	<20.0 (GPL-U/mL)
Antibody to JO-1	<1.0 NEG	<1.0
Antibody Assay, Ribosomal P Protein	<1.0 NEG	<1.0
Antibody to Antiscleroderma-70	<1.0 NEG	<1.0
Antiphospholipid Antibody Panel		
Cardiolipin antibody IgG	<2.0	(<20.0 GPL-U/mL)
Cardiolipin antibody IgA	<2.0	(<20.0 APL-U/mL)
Cardiolipin antibody IgM	<2.0	(<20.0 MPL-U/mL)
Phos. Serine AB IgG	<9	(<=30 U)
Phos. Serine AB IgM	<52	(<=30 U)
B2-Glucoprotein IgM	<2.0	(<20.0 U/mL)
B2-Glucoprotein IgA	<2.0	(<20.0 U/mL)
B2-Glucoprotein IgG	<2.0	(<20.0 U/mL)

## Discussion

This case illustrates the rare presentation of severe symptomatic hypomagnesemia manifesting primarily as isolated bilateral lower extremity weakness and tremor. Only a handful of similar cases have been described in the literature to date [6,7].

Hardwick et al. reported on a 61-year-old woman who developed acute paraparesis secondary to severe hypomagnesemia (0.2 mmol/L or 0.48 mg/dL) in the context of chronic proton pump inhibitor use and malnutrition [6]. Complete neurological recovery occurred within 48 hours of intravenous magnesium replacement.

In another case, Liamis et al.described a 42-year-old man with chronic alcoholism who presented with profound hypomagnesemia (0.36 mmol/L) during an episode of acute pancreatitis, likely due to magnesium saponification in necrotic fat, intracellular shifts from respiratory alkalosis, and hyperadrenergic state, compounded by his underlying alcohol use disorder [7]. Serum magnesium levels rapidly normalized with supplementation.

Our patient shares similarities with the case of Liamis et al., as both developed severe hypomagnesemia triggered by an acute illness resulting in gastrointestinal losses - diarrhea in our patient and pancreatitis in the other case. Chronic alcoholism was a predisposing factor for Liamis et al.'s patient, while ours had several potential contributory factors including diabetes, diuretic and proton pump inhibitor use. Magnesium repletion led to prompt resolution of neuromuscular symptoms in both instances.

The pathogenesis of neuromuscular dysfunction in hypomagnesemia involves multiple mechanisms. As a critical cofactor, magnesium facilitates neuromuscular transmission by modulating presynaptic acetylcholine release and postsynaptic depolarization [8]. Hypomagnesemia also leads to hypocalcemia by blunting parathyroid hormone (PTH) secretion and inducing end-organ PTH resistance [9]. Furthermore, magnesium depletion can cause hypokalemia via renal potassium wasting [10]. The interplay of these electrolyte disturbances leads to the clinical manifestations of muscle weakness, cramps, tremor, tetany, and arrhythmias.

In a study of patients with profound hypomagnesemia (<1.0 mg/dL), Chernow et al. noted that muscle weakness (46%), tremor (46%), tetany (18%), and positive Chvostek sign (10%) were the most prevalent neuromuscular signs, with 68% exhibiting some degree of neuromuscular irritability [11]. However, the predominance of lower extremity weakness as the initial presenting complaint, as seen in our patient, remains uncommon and intriguing. The underlying pathophysiology of this localization is uncertain but may involve a length-dependent neuropathic process [12].

The etiologies of hypomagnesemia encompass gastrointestinal losses, renal wasting, redistribution, and inadequate intake [13]. Gastrointestinal causes include diarrhea, malabsorption, nasogastric suctioning, fistulas, and proton pump inhibitor therapy. Renal losses are seen with diuretic use, hypercalcemia-mediated reduced magnesium renal reabsorption, and inherited tubulopathies like Gitelman and Bartter syndromes. Intracellular magnesium shifts occur in refeeding syndrome, post-parathyroidectomy, acute pancreatitis, and treatment of diabetic ketoacidosis [14-19]. We postulate that the hypomagnesemia in our patient was precipitated by acute gastrointestinal losses and poor dietary intake, possibly exacerbated by baseline treatment with proton pump inhibitors.

## Conclusions

Hypomagnesemia is an important and underrecognized cause of neuromuscular weakness that can mimic other neurological disorders. This case highlights the potential for hypomagnesemia to present primarily with isolated symmetric lower extremity weakness and the importance of checking serum magnesium in patients with weakness and risk factors for magnesium depletion. Prompt recognition and correction of hypomagnesemia can lead to rapid recovery of neuromuscular function and prevent complications.

Several confounding factors should be considered in such presentations, including pre-existing neuropathy (as in our patient), subtle undetected metabolic disturbances, and medication effects (e.g., proton pump inhibitors). Despite these potential contributing factors, the dramatic improvement with magnesium repletion strongly suggests a causal relationship in this case. Clinicians should maintain a high index of suspicion for hypomagnesemia in at-risk patients, particularly those with multiple comorbidities, polypharmacy, and acute gastrointestinal losses.
